# The genome sequence of the Minor Shoulder-knot, *Brachylomia viminalis *(Fabricius, 1777)

**DOI:** 10.12688/wellcomeopenres.19538.1

**Published:** 2023-06-14

**Authors:** Douglas Boyes, Clare Boyes, University of Oxford and Wytham Woods Genome Acquisition Lab, Darwin Tree of Life Barcoding collective, Wellcome Sanger Institute Tree of Life programme, Wellcome Sanger Institute Scientific Operations: DNA Pipelines collective, Tree of Life Core Informatics collective, Darwin Tree of Life Consortium

**Affiliations:** 1 UK Centre for Ecology & Hydrology, Wallingford, England, UK; 2 Independent researcher, Welshpool, Wales, UK

**Keywords:** Brachylomia viminalis, Minor Shoulder-knot, genome sequence, chromosomal, Lepidoptera

## Abstract

We present a genome assembly from an individual male *Brachylomia viminalis* (the Minor Shoulder-knot; Arthropoda; Insecta; Lepidoptera; Noctuidae). The genome sequence is 782.2 megabases in span. Most of the assembly is scaffolded into 31 chromosomal pseudomolecules, including the Z sex chromosome. The mitochondrial genome has also been assembled and is 16.15 kilobases in length. Gene annotation of this assembly on Ensembl identified 20,191 protein coding genes.

## Species taxonomy

Eukaryota; Metazoa; Eumetazoa; Bilateria; Protostomia; Ecdysozoa; Panarthropoda; Arthropoda; Mandibulata; Pancrustacea; Hexapoda; Insecta; Dicondylia; Pterygota; Neoptera; Endopterygota; Amphiesmenoptera; Lepidoptera; Glossata; Neolepidoptera; Heteroneura; Ditrysia; Obtectomera; Noctuoidea; Noctuidae; Xyleninae; *Brachylomia*; *Brachylomia viminalis* (Fabricius, 1777) (NCBI:txid988081).

## Background

*Brachylomia viminalis* (Minor Shoulder-knot) is a widespread noctuid moth of damp habitats found across the Palaearctic from Europe to Japan. In Britain, the abundance and distribution of this species has decreased significantly since the 1970s and it continues to decline ( Randle *et al.*, 2019). Consequently, ( Fox *et al.*, 2019) classified this moth as ‘near-threatened’. The adult moth is attracted to light, and also feeds at flowers during its flight season in July and August. 

The adult moth is quite small, with a forewing length of 13–15 mm, and is variable in colouration. Usually, the ground colour is light grey but darker forms occur, with melanistic forms appearing over most of its range in Britain. The specimen selected for genome sequencing here was of the light grey colour form, f. *stricta*. The two short black streaks at the base of the forewing are diagnostic and give rise to the moth’s common name: the Minor Shoulder-knot. ‘Shoulder-knots’ were fashionable in the late 1600s and were a set of ribbons on a coat in a contrasting colour. The dark streaks are thought to resemble this frippery ( Marren, 2019).

The eggs are laid either singly or in small groups on the twigs of a wide variety of willow species, predominantly grey willow ( *Salix cinerea*), and it overwinters as this stage. Eggs hatch in the spring and the larvae initially feed on terminal shoots. Later larval stages feed nocturnally and hide by day in spinnings on the leaves of terminal shoots ( Heath & Emmet, 1983). Pupation occurs in leaf litter or just under the soil surface.

The genome of *B. viminalis* was sequenced as part of the Darwin Tree of Life Project, a collaborative effort to sequence all named eukaryotic species in the Atlantic Archipelago of Britain and Ireland. Here we present a chromosomally complete genome sequence for *B. viminalis*, based on one male specimen from Wytham Woods, Oxfordshire, UK.

## Genome sequence report

**Figure 1.**
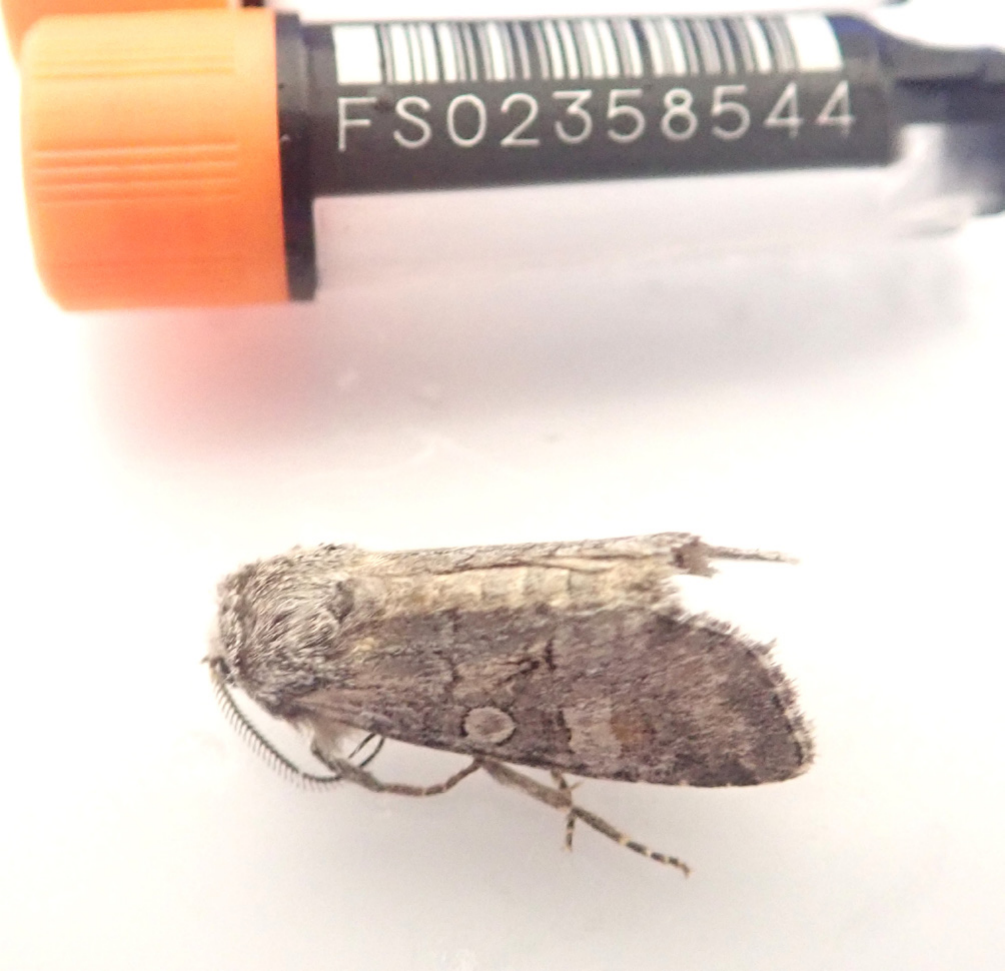
Photograph of the *Brachylomia viminalis* (ilBraVimi1) specimen used for genome sequencing.

The genome was sequenced from one male *Brachylomia viminalis* ( Figure 1) collected from Wytham Woods, Oxfordshire, UK (51.77, –1.33). A total of 51-fold coverage in Pacific Biosciences single-molecule HiFi long reads and 78-fold coverage in 10X Genomics read clouds were generated. Primary assembly contigs were scaffolded with chromosome conformation Hi-C data. Manual assembly curation corrected 22 missing joins or mis-joins and removed 3 haplotypic duplication, reducing the assembly length by 0.43% and increasing the scaffold number by 4.88%, and decreasing the scaffold N50 by 0.85%.

**Figure 2.**
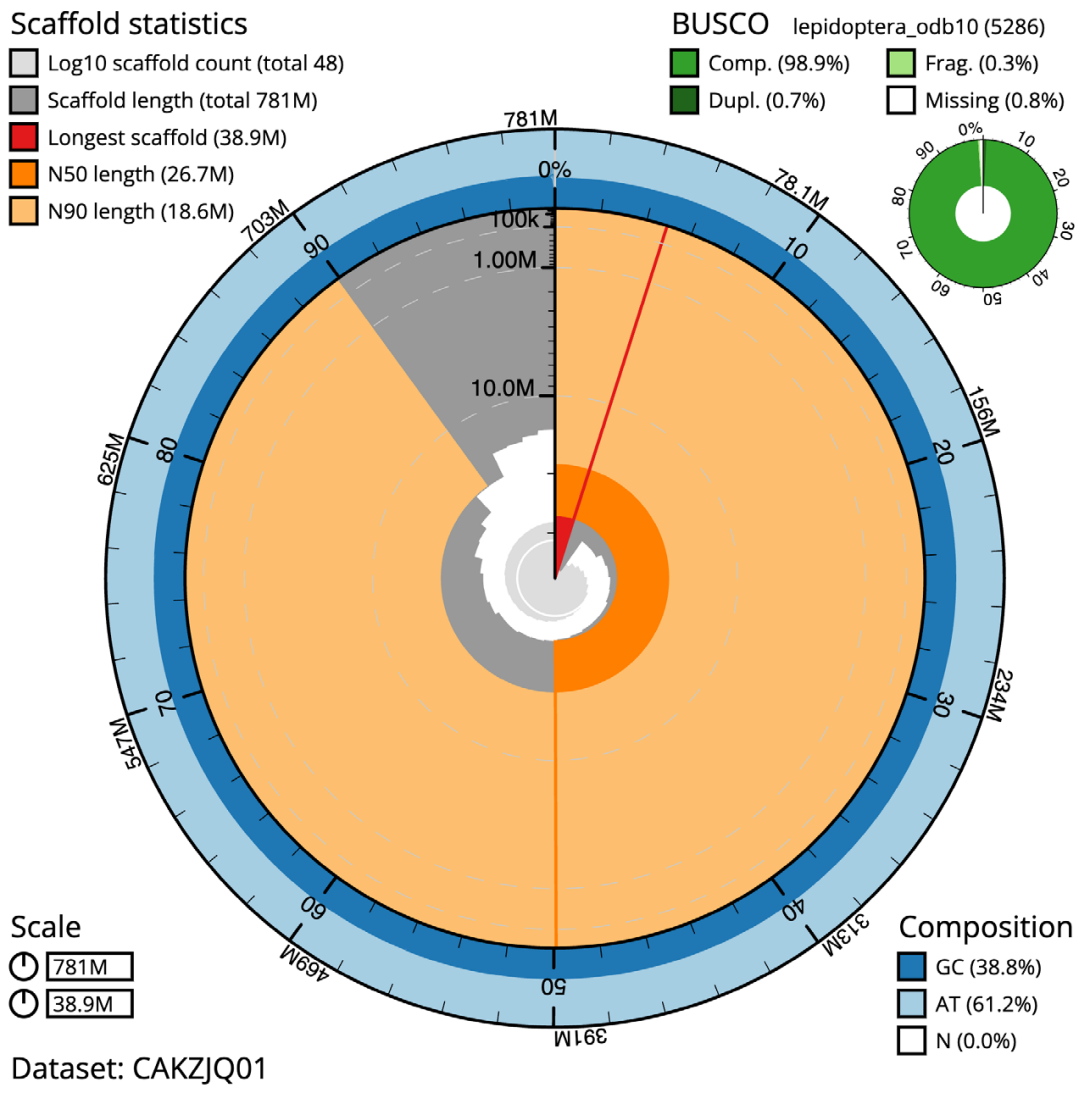
Genome assembly of *Brachylomia viminalis*, ilBraVimi1.2: metrics. The BlobToolKit Snailplot shows N50 metrics and BUSCO gene completeness. The main plot is divided into 1,000 size-ordered bins around the circumference with each bin representing 0.1% of the 781,437,518 bp assembly. The distribution of scaffold lengths is shown in dark grey with the plot radius scaled to the longest scaffold present in the assembly (38,942,460 bp, shown in red). Orange and pale-orange arcs show the N50 and N90 scaffold lengths (26,683,488 and 18,613,057 bp), respectively. The pale grey spiral shows the cumulative scaffold count on a log scale with white scale lines showing successive orders of magnitude. The blue and pale-blue area around the outside of the plot shows the distribution of GC, AT and N percentages in the same bins as the inner plot. A summary of complete, fragmented, duplicated and missing BUSCO genes in the lepidoptera_odb10 set is shown in the top right. An interactive version of this figure is available at https://blobtoolkit.genomehubs.org/view/ilBraVimi1.1/dataset/CAKZJQ01.1/snail.

The final assembly has a total length of 782.2 Mb in 42 sequence scaffolds with a scaffold N50 of 26.8 Mb ( Table 1). Most (99.9%) of the assembly sequence was assigned to 31 chromosomal-level scaffolds, representing 30 autosomes and the Z sex chromosome. Chromosome-scale scaffolds confirmed by the Hi-C data are named in order of size ( Figure 2– Figure 5; Table 2). While not fully phased, the assembly deposited is of one haplotype. Contigs corresponding to the second haplotype have also been deposited. The mitochondrial genome was also assembled and can be found as a contig within the multifasta file of the genome submission.

**Table 1.** Genome data for *Brachylomia viminalis*, ilBraVimi1.2.

Project accession data		
Assembly identifier	ilBraVimi1.2	
Species	*Brachylomia viminalis*	
Specimen	ilBraVimi1	
NCBI taxonomy ID	988081	
BioProject	PRJEB51268	
BioSample ID	SAMEA7701291	
Isolate information	ilBraVimi1	
Assembly metrics *		*Benchmark*
Consensus quality (QV)	59	*≥ 50*
*k*-mer completeness	100%	*≥ 95%*
BUSCO **	C:98.9%[S:98.2%,D:0.7%], F:0.3%,M:0.8%,n:5,286	*C ≥ 95%*
Percentage of assembly mapped to chromosomes	99.9%	*≥ 95%*
Sex chromosomes	Z chromosome	*localised homologous pairs*
Organelles	Mitochondrial genome assembled	*complete single alleles*
Raw data accessions		
PacificBiosciences SEQUEL II	ERR9127942, ERR9468771	
10X Genomics Illumina	ERR9123824–ERR9123827	
Hi-C Illumina	ERR9123823	
Genome assembly		
Assembly accession	GCA_937001585.2	
*Accession of alternate haplotype*	GCA_937001565.2	
Span (Mb)	782.2	
Number of contigs	82	
Contig N50 length (Mb)	20.7	
Number of scaffolds	42	
Scaffold N50 length (Mb)	26.8	
Longest scaffold (Mb)	39.0	
Genome annotation		
Number of protein-coding genes	20,191	
Number of gene transcripts	20,380	

* Assembly metric benchmarks are adapted from column VGP-2020 of “Table 1: Proposed standards and metrics for defining genome assembly quality” from ( Rhie *et al.*, 2021). ** BUSCO scores based on the lepidoptera_odb10 BUSCO set using v5.3.2. C = complete [S = single copy, D = duplicated], F = fragmented, M = missing, n = number of orthologues in comparison. A full set of BUSCO scores is available at https://blobtoolkit.genomehubs.org/view/ilBraVimi1.1/dataset/CAKZJQ01.1/busco.

**Figure 3.**
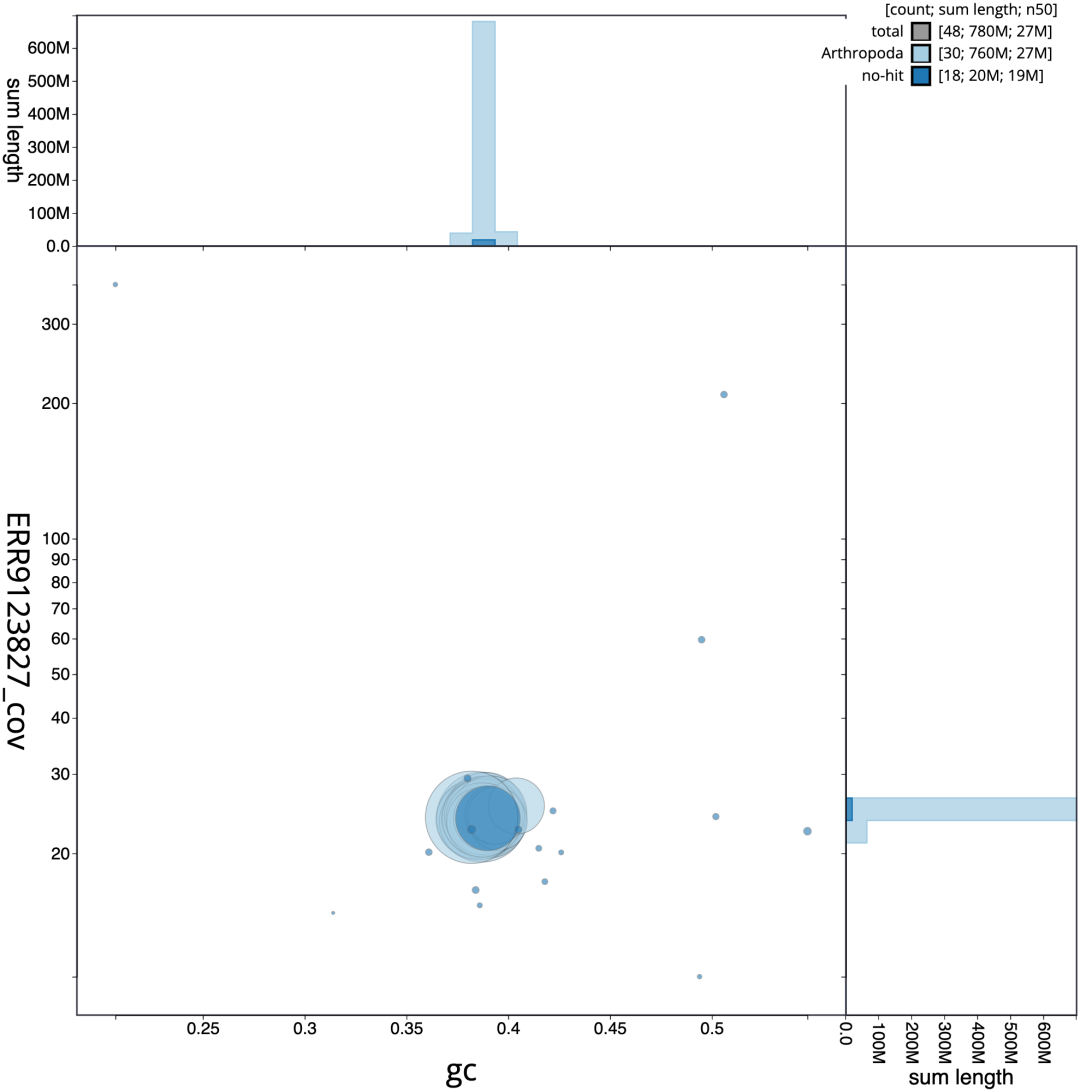
Genome assembly of *Brachylomia viminalis*, ilBraVimi1.2: BlobToolKit GC-coverage plot. Scaffolds are coloured by phylum. Circles are sized in proportion to scaffold length. Histograms show the distribution of scaffold length sum along each axis. An interactive version of this figure is available at https://blobtoolkit.genomehubs.org/view/ilBraVimi1.1/dataset/CAKZJQ01.1/blob.

**Figure 4.**
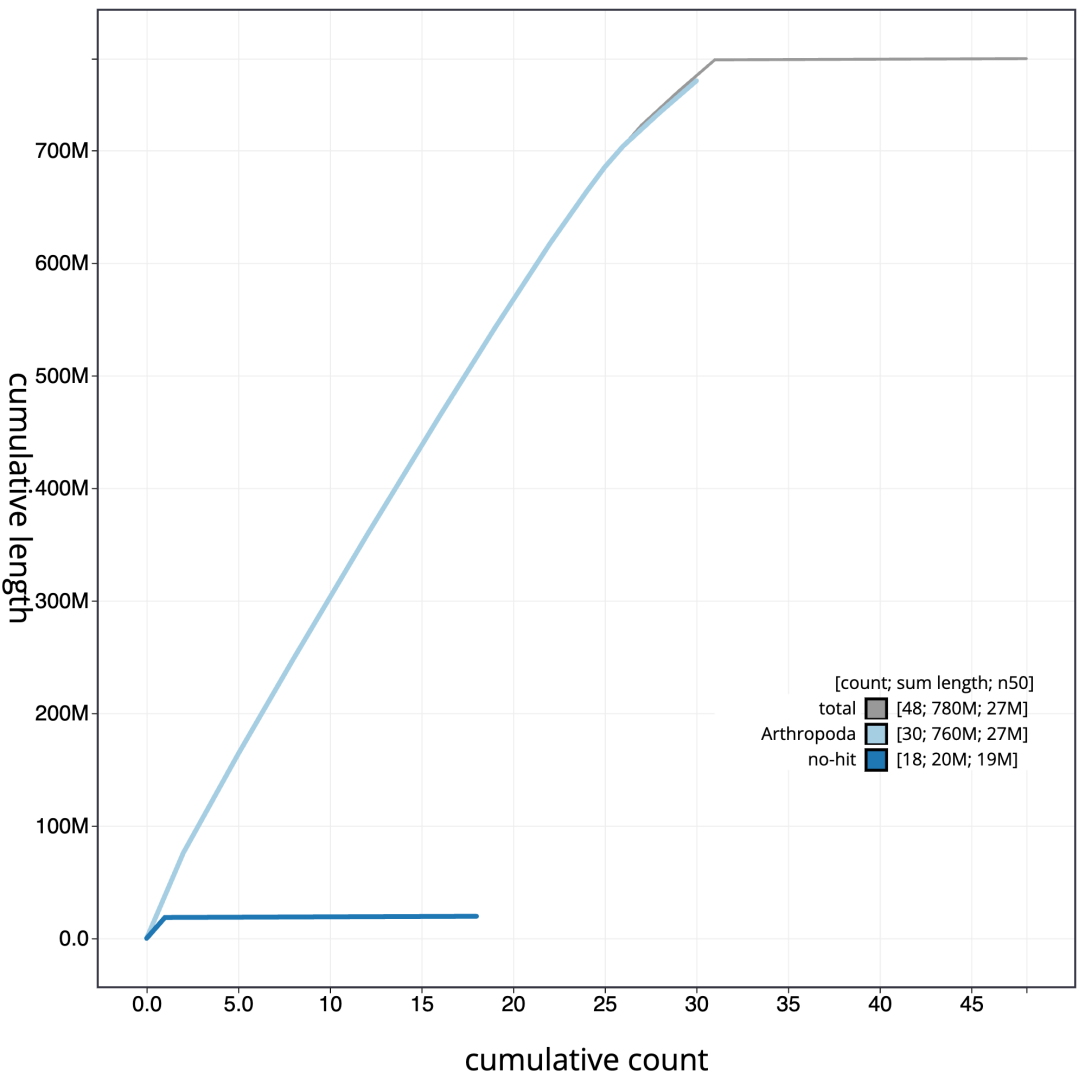
Genome assembly of *Brachylomia viminalis*, ilBraVimi1.2: BlobToolKit cumulative sequence plot. The grey line shows cumulative length for all scaffolds. Coloured lines show cumulative lengths of scaffolds assigned to each phylum using the buscogenes taxrule. An interactive version of this figure is available at https://blobtoolkit.genomehubs.org/view/ilBraVimi1.1/dataset/CAKZJQ01.1/cumulative.

**Figure 5.**
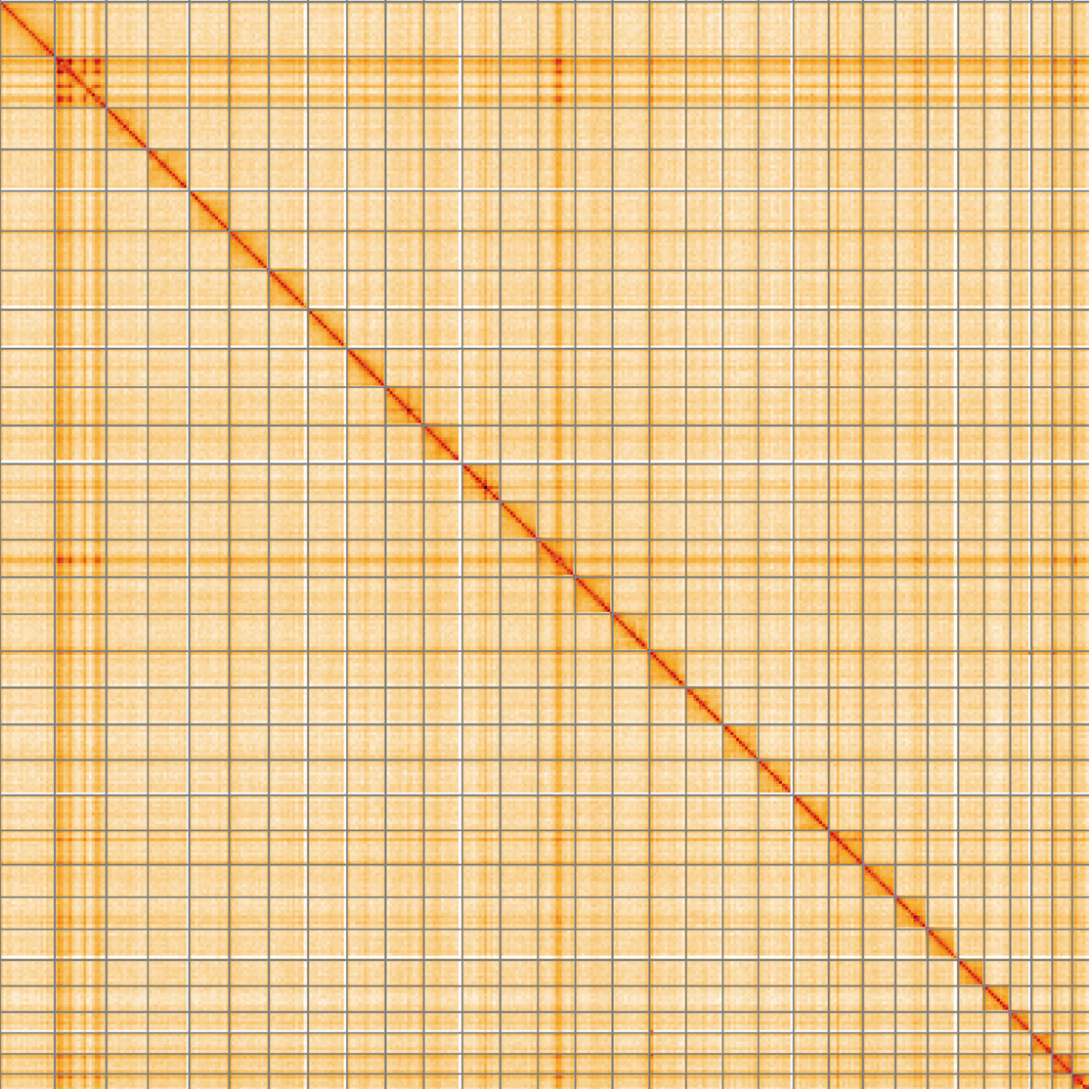
Genome assembly of *Brachylomia viminalis*, ilBraVimi1.2: Hi-C contact map of the ilBraVimi1.2 assembly, visualised using HiGlass. Chromosomes are shown in order of size from left to right and top to bottom. An interactive version of this figure may be viewed at https://genome-note-higlass.tol.sanger.ac.uk/l/?d=HQl63gQkR1yHz83QJbO33w.

**Table 2.** Chromosomal pseudomolecules in the genome assembly of *Brachylomia viminalis*, ilBraVimi1.

INSDC accession	Chromosome	Length (Mb)	GC%
OW443294.2	1	37.31	39.0
OW443295.2	2	29.84	38.5
OW443296.2	3	29.79	39.0
OW443297.2	4	28.66	38.5
OW443298.2	5	28.56	39.0
OW443299.2	6	27.45	39.0
OW443300.2	7	27.68	38.5
OW443301.2	8	27.73	39.0
OW443302.2	9	27.6	38.5
OW443303.2	10	27.47	39.0
OW443304.2	11	27.54	39.0
OW443305.2	12	26.44	39.0
OW443306.2	13	27.19	39.0
OW443307.2	14	26.8	39.0
OW443308.2	15	26.42	38.5
OW443309.2	16	25.16	38.5
OW443310.2	17	26.12	38.5
OW443311.2	18	25.59	38.5
OW443312.2	19	25.1	39.0
OW443313.2	20	25.12	38.5
OW443314.2	21	25.27	39.0
OW443315.2	22	23.09	39.0
OW443316.2	23	22.88	38.5
OW443317.2	24	22.18	39.0
OW443318.2	25	18.47	39.0
OW443319.2	26	18.57	39.0
OW443320.2	27	14.82	39.5
OW443321.2	28	15.54	39.5
OW443322.2	29	14.04	39.5
OW443323.2	30	14.14	40.5
OW443293.2	Z	38.97	38.0
OW443324.1	MT	0.02	21.0

The estimated Quality Value (QV) of the final assembly is 59 with *k*-mer completeness of 100%, and the assembly has a BUSCO v5.3.2 completeness of 98.9% (single = 98.2%, duplicated = 0.7%), using the lepidoptera_odb10 reference set ( *n* = 5,286).

Metadata for specimens, spectral estimates, sequencing runs, contaminants and pre-curation assembly statistics can be found at https://links.tol.sanger.ac.uk/species/988081.

## Genome annotation report

The *Brachylomia viminalis* genome assembly (GCA_937001585.2) was annotated using the Ensembl rapid annotation pipeline ( Table 1; https://rapid.ensembl.org/Brachylomia_viminalis_GCA_937001585.1/Info/Index). The resulting annotation includes 20,380 transcribed mRNAs from 20,191 protein-coding genes.

## Methods

### Sample acquisition and nucleic acid extraction

A male *Brachylomia viminalis* (specimen ID Ox000522, individual ilBraVimi1) was collected from Wytham Woods, Oxfordshire (biological vice-county Berkshire), UK (latitude 51.77, longitude –1.33) on 2020-06-25 using a light trap. Douglas Boyes (University of Oxford) collected and identified the specimen. The specimen was snap-frozen on dry ice.

DNA was extracted at the Tree of Life laboratory, Wellcome Sanger Institute (WSI). The ilBraVimi1 sample was weighed and dissected on dry ice with tissue set aside for Hi-C sequencing. Abdomen tissue was disrupted using a Nippi Powermasher fitted with a BioMasher pestle. High molecular weight (HMW) DNA was extracted using the Qiagen MagAttract HMW DNA extraction kit. Low molecular weight DNA was removed from a 20 ng aliquot of extracted DNA using the 0.8X AMpure XP purification kit prior to 10X Chromium sequencing; a minimum of 50 ng DNA was submitted for 10X sequencing. HMW DNA was sheared into an average fragment size of 12–20 kb in a Megaruptor 3 system with speed setting 30. Sheared DNA was purified by solid-phase reversible immobilisation using AMPure PB beads with a 1.8X ratio of beads to sample to remove the shorter fragments and concentrate the DNA sample. The concentration of the sheared and purified DNA was assessed using a Nanodrop spectrophotometer and Qubit Fluorometer and Qubit dsDNA High Sensitivity Assay kit. Fragment size distribution was evaluated by running the sample on the FemtoPulse system.

### Sequencing

Pacific Biosciences HiFi circular consensus and 10X Genomics read cloud DNA sequencing libraries were constructed according to the manufacturers’ instructions. DNA sequencing was performed by the Scientific Operations core at the WSI on Pacific Biosciences SEQUEL II (HiFi) and Illumina NovaSeq 6000 (10X) instruments. Hi-C data were also generated from head and thorax tissue of ilBraVimi1 using the Arima2 kit and sequenced on the Illumina NovaSeq 6000 instrument.

### Genome assembly, curation and evaluation

Assembly was carried out with Hifiasm ( Cheng *et al.*, 2021) and haplotypic duplication was identified and removed with purge_dups ( Guan *et al.*, 2020). One round of polishing was performed by aligning 10X Genomics read data to the assembly with Long Ranger ALIGN, calling variants with FreeBayes ( Garrison & Marth, 2012). The assembly was then scaffolded with Hi-C data ( Rao *et al.*, 2014) using YaHS ( Zhou *et al.*, 2023). The assembly was checked for contamination and corrected as described previously ( Howe *et al.*, 2021). Manual curation was performed using HiGlass ( Kerpedjiev *et al.*, 2018) and Pretext ( Harry, 2022). The mitochondrial genome was assembled using MitoHiFi ( Uliano-Silva *et al.*, 2022), which runs MitoFinder ( Allio *et al.*, 2020) or MITOS ( Bernt *et al.*, 2013) and uses these annotations to select the final mitochondrial contig and to ensure the general quality of the sequence.

A Hi-C map for the final assembly was produced using bwa-mem2 ( Vasimuddin *et al.*, 2019) in the Cooler file format ( Abdennur & Mirny, 2020). To assess the assembly metrics, the *k*-mer completeness and QV consensus quality values were calculated in Merqury ( Rhie *et al.*, 2020). This work was done using Nextflow ( Di Tommaso *et al.*, 2017) DSL2 pipelines “sanger-tol/readmapping” ( Surana *et al.*, 2023a) and “sanger-tol/genomenote” ( Surana *et al.*, 2023b). The genome was analysed within the BlobToolKit environment ( Challis *et al.*, 2020) and BUSCO scores ( Manni *et al.*, 2021; Simão *et al.*, 2015) were calculated.

Table 3 contains a list of relevant software tool versions and sources.

**Table 3.** Software tools: versions and sources.

Software tool	Version	Source
BlobToolKit	4.0.7	https://github.com/blobtoolkit/blobtoolkit
BUSCO	5.3.2	https://gitlab.com/ezlab/busco
FreeBayes	1.3.1-17-gaa2ace8	https://github.com/freebayes/freebayes
Hifiasm	0.16.1-r375	https://github.com/chhylp123/hifiasm
HiGlass	1.11.6	https://github.com/higlass/higlass
Long Ranger ALIGN	2.2.2	https://support.10xgenomics.com/genome-exome/ software/pipelines/latest/advanced/other-pipelines
Merqury	MerquryFK	https://github.com/thegenemyers/MERQURY.FK
MitoHiFi	2	https://github.com/marcelauliano/MitoHiFi
PretextView	0.2	https://github.com/wtsi-hpag/PretextView
purge_dups	1.2.3	https://github.com/dfguan/purge_dups
sanger-tol/genomenote	v1.0	https://github.com/sanger-tol/genomenote
sanger-tol/readmapping	1.1.0	https://github.com/sanger-tol/readmapping/tree/1.1.0
YaHS	yahs-1.1.91eebc2	https://github.com/c-zhou/yahs

### Genome annotation

The BRAKER2 pipeline ( Brůna *et al.*, 2021) was used in the default protein mode to generate annotation for the *Brachylomia viminalis* assembly (GCA_937001585.2) in Ensembl Rapid Release.

### Wellcome Sanger Institute – Legal and Governance

The materials that have contributed to this genome note have been supplied by a Darwin Tree of Life Partner. The submission of materials by a Darwin Tree of Life Partner is subject to the **‘Darwin Tree of Life Project Sampling Code of Practice’**, which can be found in full on the Darwin Tree of Life website here. By agreeing with and signing up to the Sampling Code of Practice, the Darwin Tree of Life Partner agrees they will meet the legal and ethical requirements and standards set out within this document in respect of all samples acquired for, and supplied to, the Darwin Tree of Life Project.

Further, the Wellcome Sanger Institute employs a process whereby due diligence is carried out proportionate to the nature of the materials themselves, and the circumstances under which they have been/are to be collected and provided for use. The purpose of this is to address and mitigate any potential legal and/or ethical implications of receipt and use of the materials as part of the research project, and to ensure that in doing so we align with best practice wherever possible. The overarching areas of consideration are:

- Ethical review of provenance and sourcing of the material
- Legality of collection, transfer and use (national and international)

Each transfer of samples is further undertaken according to a Research Collaboration Agreement or Material Transfer Agreement entered into by the Darwin Tree of Life Partner, Genome Research Limited (operating as the Wellcome Sanger Institute), and in some circumstances other Darwin Tree of Life collaborators.

## Data Availability

European Nucleotide Archive: *Brachylomia viminalis* (minor shoulder-knot). Accession number PRJEB51268; https://identifiers.org/ena.embl/PRJEB51268. ( Wellcome Sanger Institute, 2022) The genome sequence is released openly for reuse. The *Brachylomia viminalis* genome sequencing initiative is part of the Darwin Tree of Life (DToL) project. All raw sequence data and the assembly have been deposited in INSDC databases. Raw data and assembly accession identifiers are reported in Table 1.

## Author information

Members of the University of Oxford and Wytham Woods Genome Acquisition Lab are listed here: https://doi.org/10.5281/zenodo.4789928.

Members of the Darwin Tree of Life Barcoding collective are listed here: https://doi.org/10.5281/zenodo.4893703.

Members of the Wellcome Sanger Institute Tree of Life programme are listed here: https://doi.org/10.5281/zenodo.4783585.

Members of Wellcome Sanger Institute Scientific Operations: DNA Pipelines collective are listed here: https://doi.org/10.5281/zenodo.4790455.

Members of the Tree of Life Core Informatics collective are listed here: https://doi.org/10.5281/zenodo.5013541.

Members of the Darwin Tree of Life Consortium are listed here: https://doi.org/10.5281/zenodo.4783558.
