# Supplementary material for: The genome sequence of the Minor Shoulder-knot, Brachylomia viminalis (Fabricius, 1777)
Source: Wellcome Open Res. 2023 Jun 14;8:245. [Version 1] doi: 10.12688/wellcomeopenres.19538.1 (PMC10905140; doi:10.12688/wellcomeopenres.19538.1)
Supplement: Peer review history

# Reviewer report: Reviewer response for version 1

**DOI:** 10.21956/wellcomeopenres.21642.r72504

**Published:** 2024-02-29

**Authors:** Stephen Richards

**Affiliations:** 1 Baylor College of Medicine, Houston, TX, USA

This is yet another almost perfect genome assembly from the Darwin Tree of Life project from a near-threatened lepidopteran species. The genome quality is amazing, and I had no trouble getting access to it at EBI. The tree of life project is moving from success to success, and I hope they will continue to get funding to accelerate into sequencing of the rest of the UK. I hope that this reference genome will be used as a basis for population genomics to help guide how and where to best protect this species and prevent further declines. An excellent job and congratulations to all involved!

Are sufficient details of methods and materials provided to allow replication by others?

Yes

Is the rationale for creating the dataset(s) clearly described?

Yes

Are the datasets clearly presented in a useable and accessible format?

Yes

Are the protocols appropriate and is the work technically sound?

Yes

Reviewer Expertise:

Arthropod reference genome generation and analysis, general genomics.

I confirm that I have read this submission and believe that I have an appropriate level of expertise to confirm that it is of an acceptable scientific standard.

# Reviewer report: Reviewer response for version 1

**DOI:** 10.21956/wellcomeopenres.21642.r60400

**Published:** 2023-07-10

**Authors:** Faezah Mohd Salleh

**Affiliations:** 1 Department of Biosciences, Universiti Teknologi Malaysia, Skudai, Johor, Malaysia

Thank you for inviting me to review this genome assembly work. An important contribution to the insect community.

 The paper is clearly written. All metadata, genome statistics, and related links have been provided and are accessible. The research flow has been described thoroughly and is highly reproducible.

 I do not have further suggestions.

Are sufficient details of methods and materials provided to allow replication by others?

Yes

Is the rationale for creating the dataset(s) clearly described?

Yes

Are the datasets clearly presented in a useable and accessible format?

Yes

Are the protocols appropriate and is the work technically sound?

Yes

Reviewer Expertise:

Biodiversity mitogenomics

I confirm that I have read this submission and believe that I have an appropriate level of expertise to confirm that it is of an acceptable scientific standard.
